# Associations among Bone Mineral Density, Physical Activity and Nutritional Intake in Middle-Aged Women with High Levels of Arterial Stiffness: A Pilot Study

**DOI:** 10.3390/ijerph17051620

**Published:** 2020-03-03

**Authors:** Kanako Hamaguchi, Toshiyuki Kurihara, Masahiro Fujimoto, Koji Sato, Motoyuki Iemitsu, Takafumi Hamaoka, Kiyoshi Sanada

**Affiliations:** 1 College of Sport and Health Science, Ritsumeikan University, Shiga 525-8577, Japan; gr0129xx@ed.ritsumei.ac.jp (K.H.); t-kuri@mti.biglobe.ne.jp (T.K.); iemitsu@fc.ritsumei.ac.jp (M.I.); 2 National Institute of Advanced Industrial Science and Technology, Chiba 277-0882, Japan; masahiro-fujimoto@aist.go.jp; 3 Graduate School of Human Development and Environment, Kobe University, Hyogo 657-8501, Japan; sato712@people.kobe-u.ac.jp; 4 Department of Sports Medicine for Health Promotion, Tokyo Medical University, Nishi-Shinjuku 6-7-1, Shinjuku-ku, Tokyo 160-0023, Japan; kyp02504@nifty.com

**Keywords:** osteoporosis, nitric oxide, sedentary, hypertension, walking, oleic acid, linoleic acid, inflammation, antioxidant, postmenopausal women

## Abstract

There is little consensus regarding the impacts of physical activity and nutrient intake on bone mineral density (BMD) in subjects with high or low levels of arterial stiffness. This study was performed to investigate whether physical activity and nutrient intake are associated with BMD in middle-aged women with high levels of arterial stiffness. The study population consisted of middle-aged women aged 40–64 years (*n* = 22). BMD was assessed by dual-energy X-ray absorptiometry. Carotid-femoral pulse wave velocity (cf-PWV) was used as an indicator of arterial stiffness. Subjects were divided into two groups by median cf-PWV. Physical activity in free-living conditions was evaluated using a triaxial accelerometer. Nutrient intake was also measured using the brief-type self-administered diet history questionnaire. In the High-PWV group, BMD showed a significant negative correlation with age. Using a partial correlation model, BMD was associated with the number of steps and unsaturated fatty acid intake in the High-PWV group. These results suggest that BMD in middle-aged women with high levels of arterial stiffness may be associated with both the number of steps and nutritional intake. Recommendations of physical activity and nutritional intake for the prevention of osteopenia should include consideration of arterial stiffness.

## 1. Introduction

Osteoporosis, a chronic disease characterised by reduced bone mineral density (BMD), is one of the most prevalent factors contributing to fractures, mortality and mobility limitation in the elderly [1], and the prevalence in women is two- to sixfold higher than in men [2,3]. Treating established osteoporosis is difficult because drugs available for the prevention of osteoporosis may have long-term adverse effects and are often expensive. It is essential to prevent the development of this disease in the aging society. Age-related reductions in BMD are generally accelerated in women after menopause [2,3]. Age-related bone reduction occurs at a higher rate in middle age than in old age. Therefore, it is necessary to prevent bone loss in middle age. In general, age-related reductions in BMD are multifactorial. Therefore, optimisation of lifestyle-related factors known to influence BMD is an important strategy. Physical activity and adequate nutrition are key lifestyle approaches for the prevention of osteoporosis [4,5,6,7,8]. Although several studies have reported protective factors for BMD in middle-aged women, these findings remain controversial. Wolff et al. investigated the randomized controlled trials and nonrandomized controlled trials on the effects of exercise training programs on bone in women. As a result, the treatment effects for the nonrandomized controlled trials were almost twice as high as those for the randomized controlled trials [9]. This evidence suggests that exercise is most effective if they are individually tailored and include appropriate types and doses. 

Arterial stiffness and Atherosclerosis can be focused as one of the factors of these disagreements. Because recent studies also suggested that there is potential for cross-talk between arterial systems and bone metabolism [10,11,12]. Atherosclerosis is a chronic inflammatory disease characterised by the accumulation of oxidised lipoproteins in the arterial wall [13,14]. Relations between endothelial dysfunction, cholesterol elevation, inflammation, thrombosis and atherogenesis have been established [15]. For example, arterial stiffness and Atherosclerosis have been implicated as a potential contributor to bone health due to the potential effects of nitric oxide (NO) [16,17,18,19,20] and atherogenic materials (e.g., oxidative stress) [21,22] on osteocyte function. Therefore, reducing arterial stiffness may also influence bone health in women with progressing arteriosclerosis.

Physical activity and nutrient intake are modifiable factors that influence arterial stiffness. Habitual physical activity has a positive effect on arterial distensibility [19,23,24,25,26,27]. Boyle et al. provided evidence for the deleterious impact of short-term reductions in daily activity on vascular health [25], as well as evidence that arterial stiffness increases with age in sedentary women. Significant age-related increases in arterial stiffness, however, are not observed in women with high levels of physical activity [23]. Light physical activity has also been shown to be associated with attenuation of arterial stiffening in sedentary individuals [26].

Kahwati et al. reported that neither vitamin D, calcium, nor combined supplementation was associated with reduced fracture incidence in healthy adults without known nutrient deficiency [4]. Avenell et al. evaluated vitamin D for preventing fractures and reported that vitamin D was beneficial in populations at high-risk of fracture but not low-risk populations [28]. These studies indicated that nutrients may have effects only in specific populations. Therefore, it is important to assess the differences in nutritional effects on BMD in groups with different characteristics. Kruger and Horrobin reported that essential fatty acid-deficient animals develop severe osteoporosis coupled with increased renal and arterial calcification. They suggested that the interaction between essential fatty acids and calcium or vitamin D metabolism may be associated with osteoporosis and ectopic calcification [29]. Many studies have focused on the quantity and type of dietary fat, as well as its effects on arterial stiffness [30,31,32,33,34]. Cross-sectional data suggest that saturated fats adversely affect vascular function, whereas polyunsaturated fatty acids (PUFAs) have beneficial effects [33]. Hu et al. suggested that dietary strategies are effective in preventing arterial disease if they include a substitution of saturated fatty acids and increased consumption of unsaturated fatty acids from fish or plant sources [30]. Physical activity and nutrient intake are considered to be modifiable factors that influence arterial stiffness.

There is limited consensus, however, on the effects of physical activity and nutrient intake on individuals with high or low levels of arterial stiffness. The effects of these types of physical activity and nutrition intake on BMD remain unclear. This pilot study was performed to investigate whether physical activity and nutrient intake are associated with BMD in middle-aged women with high levels of arterial stiffness. 

## 2. Materials and Methods

### 2.1. Subjects

Middle-aged women younger than 65 years of age were recruited from local communities for this study. Data related to sociodemographic characteristics (date of birth), medical history (hypertension, hyperlipidaemia, diabetes mellitus, stroke, heart disease, renal failure and arthralgia), current smoking status and alcohol intake and current medications (name and dosage) were collected during the screening visit. Women with any medical considerations, including taking specific drugs or supplements (osteoporosis, hypertension, hyperlipidaemia, diabetes mellitus, stroke, heart disease, renal failure, arthralgia and history of fracture in the previous 12 months) that could affect bone metabolism were excluded. The study population included seven subjects with SBP > 140 mmHg, but they had not received medical treatment. Ten women fulfilled the exclusion criteria. All subjects provided written informed consent after being informed of the benefits and risks of the investigation. This study was approved by the Ethics Committee of Ritsumeikan University (Approval Number: BKC-IRB-2012-032). All participants reviewed and signed informed consent forms in accordance with the Declaration of Helsinki. 

### 2.2. Dual-Energy X-Ray Absorptiometry

Body composition was assessed by dual-energy X-ray absorptiometry (DXA) using enCORE software (Lunar Prodigy; GE Healthcare, Buckinghamshire, UK). Subjects fasted overnight and did not perform any exercise in the morning before measurements were taken. In this study, BMD values were derived from total-body DXA scans. Total-body BMD is not an appropriate surrogate for diagnosing osteoporosis or assessing fracture risk [35] because the relationship between total-body BMD and fracture risk has not been adequately defined. On the other hand, Looker et al. suggested that these data provide a unique opportunity to assess differences in BMD between groups across the entire skeleton [36], and it may be useful when investigating the effects of nutrient intake and physical activity, such as aerobic exercise or walking. Total-body and regional BMD, fat mass and fat-free mass (FFM) were analysed. All scans were performed and analysed by a single trained and licensed technician who was blinded to participant group allocation. Body mass index (BMI) was determined by dividing body weight in kilograms (kg) by height in meters squared (m^2^). Skeletal muscle mass index was calculated by dividing the appendicular fat-free mass (FFM) in kilograms (kg) by height in meters squared (m^2^). 

### 2.3. Measurement of Arterial Stiffness and Blood Pressure

Pulse wave velocity (PWV) is considered to be an indirect indicator of arterial stiffness, reflecting vascular damage. Subjects rested for 15 minutes in the supine position before carotid-femoral PWV (cf-PWV), brachial-ankle PWV (ba-PWV) and blood pressure measurements were taken. The cf-PWV, ba-PWV and blood pressure were measured simultaneously using a vascular testing device (form PWV/Ankle brachial index (ABI); Omron Colin, Kyoto, Japan). The cf-PWV was measured using applanation tonometry, with an array of 15 transducers (form PWV/ABI), as described previously [37,38,39]. The distance travelled by each pulse wave was assessed by a random zero-length measurement on the surface of the body using a non-elastic tape measure. Pulse wave transit time was determined by measuring the time delay between the proximal and distal foot waveforms. The foot of the wave was identified as the commencement of the sharp systolic upstroke, which was detected automatically. In this study, the coefficient of variation for interobserver reproducibility of cf-PWV was 4.7%. The mean values of systolic and diastolic blood pressure in the right and left arms were calculated for analysis. 

### 2.4. Analysis of Blood Samples

Blood was drawn from subjects in the seated position. Fasting (>12 h) blood samples were collected by venipuncture in tubes with or without ethylene diamine tetraacetic acid (for plasma or serum). Blood samples were centrifuged at 1500 rpm for 15 min and were stored at −20 °C. The serum concentration of triglycerides was determined using commercial kits (Mitsubishi Chemical Medience, Tokyo, Japan). Serum high-density lipoprotein cholesterol (HDL-C) was measured by an enzymatic method (Mitsubishi Chemical Medience). Fasting plasma glucose (FPG) was measured by the glucose dehydrogenase method. Whole-blood glycohemoglobin A1c (HbA1c) was measured by an enzymatic method (Glycohemoglobin A1c kit; Mitsubishi Chemical Medience).

### 2.5. Evaluation of Physical Activity

The duration and intensity of physical activity in free-living conditions were evaluated using a triaxial accelerometer (Active Style Pro HJA-350IT; Omron Healthcare; Muko, Kyoto, Japan) [40,41]. The accelerometer was attached to the waist of each subject during waking hours for 7 days, including both weekdays and weekends. Valid data were those that included at least 10 hours of wearing time. The criterion for acceptable pedometer data was that data were collected at least three days per week, including at least one weekday and one weekend day. The Omron Active Style Pro HJA-350IT measures activity intensity over a 60-s period and estimates the metabolic equivalents of the task (METs). Steps and total minutes spent in moderate to vigorous physical activity (MVPA; ≥ 3 METs) taken per day were used as physical activity outcome measures. 

### 2.6. Nutrient Intake

Nutrient intake was measured using the brief-type self-administered diet history questionnaire (BDHQ), which is a fixed-portion questionnaire that assesses dietary intake during the previous month [42,43]. The unadjusted intakes of energy and nutrients measured by the BDHQ were calculated using an ad hoc computer algorithm based on the Standard Tables of Food Composition in Japan. Intakes of saturated fatty acids, monounsaturated fatty acids (MUFAs), PUFAs, *n*-3 fatty acids and *n*-6 fatty acids were calculated in grams per day using the food list section of the BDHQ. Total energy was calculated in kilocalories per day from BDHQ responses. Questionnaires indicating an extreme energy intake of greater than two standard deviations were considered to be invalid and were excluded. 

### 2.7. Other Variables

Covariates were measured at the same time as the DXA assessment and included age (years) and height (cm). Height without shoes was measured using a stadiometer.

### 2.8. Statistical Analysis

Outcome measures included BMD (arm, spine, pelvis, leg and total body), physical activity and nutritional intake. Fat intake was defined as SFA, MUFA and PUFA in grams per day. Fat intake was then adjusted for total energy intake with the residual method [44]. In this method, energy-adjusted nutrient intakes are computed as the residuals from the regression model with total caloric intake as the independent variable and absolute nutrient intake as the dependent variable. Potential differences between the Low- and High-PWV groups were assessed using the independent *t*-test. All measurements and calculated values are expressed as the means ± standard deviation. Correlation analyses of total-body and regional BMD with study variables were performed using Pearson’s correlation analyses. Partial correlations were adjusted for age and BMI and used to examine the relationships between BMD, physical activity and nutritional intake. All analyses were performed using SPSS (version 19; IBM, Chicago, IL, USA). Statistical significance was set at α < 0.05.

## 3. Results

We assessed cf-PWV to evaluate arterial stiffness (median cf-PWV = 1053 cm/s). All subjects were classified into two groups by cf-PWV (Low- and High-PWV groups). Descriptive characteristics for Low- and High-PWV groups, as well as all subjects, are shown in Table 1, Table 2 and Table 3. Clinical features were similar for Low- and High-PWV groups, except for arterial stiffness parameters (Table 1). Findings regarding physical activity and nutrient intake were similar in both Low- and High-PWV groups, except for SFA intake (Table 2 and Table 3).

Table 4 summarises the results of correlation analysis regarding the relationships between clinical features and BMD. Total-body, arm and leg BMD were negatively associated with age in all subjects (r = −0.44, r = −0.60, r = −0.51, respectively). Similar results were observed in the High-PWV group. In all subjects, the spine and pelvis BMD were associated with BMI (r = 0.46, r = 0.49, respectively). 

In this study, age was associated with total and non-locomotive MVPA in all subjects (r = 0.49, r = 0.46, respectively) and BMI was associated with locomotive MVPA (r = 0.52). Similar results were observed in the High-PWV group. BMI and body fat were associated with total MVPA (r = 0.66, r = 0.68, respectively). In the Low-PWV group, age was associated with total and non-locomotive MVPA (r = 0.64, r = 0.63, respectively, *p* < 0.05). To exclude the possible effect of age on physical activity, we performed partial correlation analysis.

In the High-PWV group, using a partial correlation model, the number of steps taken showed a significant association with leg BMD (r = 0.67) when adjusted for age and BMI (Table 5). Significant associations were observed between MUFA and total-body, spine and pelvis BMD (r = 0.82, r = 0.88, r = 0.72, respectively). Similarly, PUFA results were associated with total-body, arm, spine and pelvis BMD (r = 0.80, r = 0.77, r = 0.88, r = 0.74, respectively). Moreover, α-tocopherol equivalents (Eq) results were associated with total-body, arm, and spine BMD (r = 0.68, r = 0.71, r = 0.72, respectively). 

## 4. Discussion

This study was performed to investigate the associations of physical activity and nutrient intake with BMD in middle-aged women with high levels of arterial stiffness. To our knowledge, there have been no previous studies directly comparing physical activity and nutrient intake in middle-aged women with low and high levels of arterial stiffness.

The main results of our study were as follows: 1) in the High-PWV group, total-body, arm and leg BMD were negatively associated with age; 2) in the High-PWV group, a positive partial correlation was found between the number of steps and leg BMD after adjusting for age and BMI; 3) in the High-PWV group, partial correlations were observed between MUFA, PUFA, α-tocopherol Eq intake and BMD.

In the High-PWV group, BMD was negatively associated with age. Previous studies have demonstrated that early postmenopausal women can be “fast bone losers” (bone loss > 3% annually) or “slow bone losers” (bone loss ≤ 3% annually) due to differences in basal bone turnover [45,46]. Sumino et al. reported that PWV was significantly correlated with BMD, but not with age or years since menopause, based on the results of multivariate regression analysis [47]. In addition, inflammatory cytokines derived from atherosclerosis can activate osteoclasts and promote bone resorption [21,48], and endothelial dysfunction due to arteriosclerosis decreases nitric oxide production, which promotes bone formation [16,17,18,19,20]. These results suggest that middle-aged women with high levels of arterial stiffness may have a fast rate of bone loss at menopause because the effects of arteriosclerosis and cytokines were added to the decrease in BMD with aging.

Some studies have highlighted a significant association between the severity of atherosclerosis and BMD [47,49], with the association being specific to the site of the lesion [50]. Among postmenopausal women, daily administration of nitroglycerine ointment (NO donor) has been shown to affect arterial dilation and blood flow, thus increasing bone formation and decreasing bone resorption [51]. These findings suggest that among women with high levels of arterial stiffness, one of the causes of bone loss is reduced blood flow, and it is predicted that BMD can be maintained by improving blood flow.

Evans et al. reported that a 9-month walking program with protein supplementation had no effect on BMD in postmenopausal women [52]. Similarly, Cavanaugh and Cann showed that a 52-week walking exercise program did not prevent the loss of BMD in early postmenopausal women [53]. In general, walking activity provides only a slight increase in load on the bone compared to resistance training, and therefore this type of exercise is less effective in promoting bone formation [54,55]. On the other hand, the positive effects of walking exercise have also been tested in more specific populations. Yamazaki et al. reported that 12 months of moderate walking exercise in postmenopausal women with osteopenia or osteoporosis had a positive effect on maintaining BMD and reducing markers of bone resorption [56]. Kitagawa et al. reported that the number of walking steps showed significant positive correlations with bone parameters and a significant decrease in bone resorption markers in elderly women [57]. These studies suggested that the mechanism underlying the positive effect of walking exercise on BMD appears to involve the suppression of bone resorption. The observations of this study suggested that walking exercise may maintain or slow the loss of BMD in middle-aged women with high levels of arterial stiffness due to improved blood flow and reduced bone resorption. 

In the High-PWV group, partial correlations were observed between unsaturated fatty acids (MUFA, PUFA) and α-tocopherol eq intake and BMD. These observations can be explained by antioxidant and anti-inflammatory effects.

Saturated fatty acids, levels of which were significantly elevated in the PWV-High group, have been suggested to be atherogenic through induction of endothelial dysfunction [58,59]. Numerous reports have indicated increased oxidative stress and chronic inflammation in patients with atherosclerosis [60,61,62,63]. Inflammatory cytokines (TNF, IL-6, IL-1), which contribute to inflammatory responses, have been reported to promote osteoclast differentiation [64,65,66,67], and oxidative stress was shown to inhibit osteogenic bone formation and differentiation maturation [21]. The PWV-High group may have elevated levels of oxidative stress and inflammatory cytokines that resulted in increased bone resorption and decreased bone formation.

Oleic acid, a typical monounsaturated fatty acid, and linoleic acid, an *n*-6 polyunsaturated fatty acid, have been shown to produce anti-inflammatory cytokines (IL-4, IL-10, IL-13) [33,68]. Moreover, nitroalkenes generated from oleic acid and linoleic acid under conditions of oxidative stress can exert anti-inflammatory effects [69]. 

Zhang et al. investigated whether antioxidant intake was associated with risk of fracture and whether this association was modified by the smoking status that induces excessive oxidative stress. The results indicated that vitamin E (antioxidant) intake was associated with reduced risk of fracture in ever smokers but not in never smokers [70]. Similarly, Melhus et al. examined whether the dietary intake of antioxidant vitamins may modify the increased fracture risk associated with smoking. The odds ratio for fracture among recent smokers with a low intake of vitamin E was 3.0. In contrast, the OR decreased to 1.1 with a high intake of vitamin E. On the other hand, the influence of vitamin E was less pronounced in former smokers [71]. 

In addition, Takeshima et al. reported that osteoblast differentiation is inhibited by oxidative stress and rescued by antioxidant treatment with vitamin E [72]. These studies suggest a role of oxidant stress in the adverse effects on the BMD, and that insufficient dietary vitamin E intake substantially increases the risk of fracture in people with high levels of oxidative stress, whereas a more adequate intake seems to have a protective effect. The above results suggest that UFA and vitamin E may suppress osteoclast differentiation and activity in the PWV-High group, but not the PWV-Low group, because the role of UFA and vitamin E intake in BMD may be modified by oxidative stress.

The major limitations of this study were the small sample size, although the nutritional intake and physical activity of this study may reflect mean values for present Japanese middle-aged women [42,73,74,75,76,77]. Future studies should investigate the associate physical activity and nutritional intake to the bone mineral density of women with differences in arterial stiffness with a larger sample size to evaluate the validity. Second, nutrient intake was self-reported by the participants. Although BDHQ is the common dietary assessment method used in Japanese studies, it contains a limited list of food items and is difficult to provide accurate reports of food consumption for the general population. Therefore, nutrient intake was adjusted for total energy intake with the residual method. Furthermore, oxidative stress, antioxidant and inflammatory cytokines, as well as nutrients in the blood have not been investigated. It is unclear whether concentration in blood or dietary intake of these materials is more important in middle-aged women. Moreover, nutrient interactions may also affect the results. Further well-designed studies are needed to understand the mechanism and evaluate the effects of nutrient intake on osteoporosis. Finally, a critical cf-PWV cut-off value of 1000 cm/s has been adopted for hypertension management or for prediction of the occurrence of cardiovascular events in elderly people [78,79,80]. However, the threshold at which arterial stiffness alters osteoporosis has not been established. Further studies are required to estimate the cut-off value at which arterial stiffness impacts osteoporosis in postmenopausal women. 

## 5. Conclusions

An understanding of vascular-related bone loss may be necessary for effective and efficient prevention and treatment of osteopenia in middle-aged women. The recommendation of physical activity and nutritional intake interventions for the prevention of osteopenia should include consideration of arterial stiffness. 

## Figures and Tables

**Table 1:** Subject characteristics at baseline for the whole study group and for Low- and High-PWV groups separately.

Varieties	All (n=22)			Low (n=11)			High (n=11)			*p*	
	Mean ± SD			Mean ± SD			Mean ± SD				
Age (yrs)	55.1 ± 6.2			55.4 ± 5.4			54.8 ± 7.1			0.84	
BMI (kg/m^2^)	20.0 ± 1.9			20.2 ± 1.7			19.8 ± 2.1			0.57	
Body fat percentage (%)	26.4 ± 6.0			27.2 ± 4.7			25.7 ± 7.3			0.58	
SMI (kg/m^2^)	5.7 ± 0.5			5.8 ± 0.3			5.7 ± 0.7			0.60	
BMD (g/cm^2^)											
Total body	1.036 ± 0.107			1.078 ± 0.103			0.994 ± 0.099			0.07	
Arm	0.734 ± 0.068			0.745 ± 0.061			0.722 ± 0.075			0.44	
Spine	0.941 ± 0.149			0.979 ± 0.155			0.903 ± 0.140			0.24	
Pelvis	0.989 ± 0.128			1.034 ± 0.106			0.945 ± 0.138			0.10	
Leg	1.083 ± 0.122			1.132 ± 0.103			1.033 ± 0.124			0.06	
Atherosclerosis parameter											
cf-PWV (cm/s)	1071 ± 145			953 ± 70			1189 ± 94			<0.01 **	
ba-PWV (cm/s)	1472 ± 375			1251 ± 219			1693 ± 374			<0.01 **	
SBP (mmHg)	126 ± 25			111 ± 17			142 ± 22			<0.01 **	
DBP (mmHg)	75 ± 14			67 ± 11			82 ± 12			<0.01 **	
Biochemical parameter											
Total cholesterol (mg/dl)	228 ± 37			221 ± 38			235 ± 36			0.38	
Triglyceride (mg/dl)	77 ± 35			74 ± 30			80 ± 41			0.66	
HDL cholesterol (mg/dl)	87 ± 20			86 ± 21			88 ± 19			0.87	
LDL cholesterol (mg/dl)	125 ± 35			120 ± 37			131 ± 34			0.46	
HDL percentage (%)	39 ± 10			40 ± 10			38 ± 9			0.68	
Blood sugar (mg/dl)	88 ± 6			87 ± 4			89 ± 8			0.39	
HbA1c (%)	5.1 ± 0.3			5.0 ± 0.3			5.2 ± 0.4			0.24	

Data are presented as means ± SD. BMI, body mass index; SMI, skeletal mass index; BMD, bone mineral density; SBP, systolic blood pressure; DBP, diastolic blood pressure; cf-PWV, carotid-femoral pulse wave velocity; ba-PWV, brachial-ankle pulse wave velocity. Potential differences between the Low- and High-PWV groups were assessed using the independent t-test. **, *p* < 0.01.

**Table 2:** Physical activity parameters for the whole study group and Low- and High-PWV groups separately.

Varieties	All (n=22)			Low (n=11)			High (n=11)			*p*
	Mean ± SD			Mean ± SD			Mean ± SD			
Accelerometry parameter (/day)										
Total MVPA ( MET * hour)	5.1 ± 2.4			5.7 ± 2.6			4.6 ± 2.1			0.30
Locomotive	2.0 ± 0.8			2.1 ± 0.7			1.9 ± 1.0			0.47
Non-Locomotive	3.1 ± 2.2			3.6 ± 2.6			2.7 ± 1.8			0.37
Steps	7349 ± 1663			7467 ± 1238			7231 ± 2061			0.75

Data are presented as means ± SD. MVPA, moderate-to-vigorous physical activity. Potential differences between the Low- and High-PWV groups were assessed using the independent t-test. *, *p* < 0.05.

**Table 3:** Nutritional parameters for the whole study group and Low- and High-PWV groups separately.

Varieties	All (n=22)			Low (n=9)			High (n=11)			*p*
	Mean ± SD			Mean ± SD			Mean ± SD			
Nutritional parameters (/day)										
Protein (g)	68.6 ± 11.3			70.4 ± 14.9			67.2 ± 8.1			0.55
Fat (g)	53.5 ± 8.5			50.9 ± 8.5			55.7 ± 7.0			0.18
SFA	14.5 ± 3.7			12.7 ± 2.8			15.9 ± 3.3			0.03 *
MUFA	19.1 ± 3.5			18.2 ± 4.1			19.8 ± 2.5			0.29
PUFA	12.7 ± 2.2			12.9 ± 2.2			12.6 ± 2.2			0.75
n-3 fatty acids	2.6 ± 0.6			2.8 ± 0.7			2.4 ± 0.5			0.21
n-6 fatty acids	10.1 ± 1.9			10.1 ± 1.9			10.1 ± 1.8			0.96
Carbohydrate (g)	238 ± 22			242 ± 28			234 ± 17			0.45
Sodium (mg)	3737 ± 606			3915 ± 673			3592 ± 529			0.24
Potassium (mg)	2864 ± 711			3011 ± 925			2744 ± 509			0.42
Calcium (mg)	564 ± 148			580 ± 194			550 ± 105			0.67
Magnesium (mg)	261 ± 62			278 ± 81			246 ± 40			0.26
Phosphorus (mg)	1064 ± 201			1103 ± 268			1031 ± 137			0.45
Iron (mg)	8.3 ± 2.1			9.0 ± 2.5			7.7 ± 1.5			0.18
Zinc (mg)	8.1 ± 0.9			8.5 ± 1.0			7.9 ± 0.8			0.17
Copper (mg)	1.2 ± 0.2			1.2 ± 0.2			1.1 ± 0.2			0.10
Manganese (mg)	3.5 ± 0.8			3.5 ± 0.8			3.4 ± 0.9			0.80
β-carotene eq (mg)	4842 ± 2377			5739 ± 3025			4107 ± 1532			0.13
Vitamin D (µg)	14.2 ± 8.7			17 ± 12			12 ± 4			0.24
α-tocopherol eq (mg)	9.3 ± 1.9			9.7 ± 2.1			9.0 ± 1.7			0.41
Vitamin K (μg)	324 ± 154			382 ± 168			277 ± 132			0.13
Vitamin B1 (mg)	0.8 ± 0.1			0.9 ± 0.2			0.8 ± 0.1			0.68
Vitamin B2 (mg)	1.4 ± 0.3			1.4 ± 0.4			1.4 ± 0.3			0.93
Niacin (mg)	17.3 ± 4.1			18.0 ± 5.4			16.7 ± 2.7			0.49
Vitamin B6 (mg)	1.4 ± 0.3			1.5 ± 0.4			1.3 ± 0.3			0.27
Vitamin B12 (µg)	8.8 ± 4.3			9.9 ± 5.6			7.8 ± 2.6			0.29
Folic acid (µg)	401 ± 130			444 ± 158			366 ± 99			0.19
Pantothenic acid (mg)	6.7 ± 1.2			6.9 ± 1.5			6.5 ± 1.0			0.56
Vitamin C (mg)	146 ± 57			158 ± 69			136 ± 46			0.41
Suger (g)	13.4 ± 6.6			11.0 ± 6.1			15.3 ± 4.2			0.08
Alcohol (g)	5.4 ± 10.4			10.8 ± 12.8			0.9 ± 1.4			0.02
Genistein (mg)	20.8 ± 14.2			24.7 ± 14.5			17.6 ± 13.6			0.27

Data are presented as means ± SD. SFA, saturated fatty acid; MUFA, onounsaturated fatty acid; PUFA, polyunsaturated fatty acid; Eq, equivalents. Potential differences between the Low- and High-PWV groups were assessed using the independent t-test. *, *p* < 0.05.

**Table 4:** Correlation coefficients between analysed characteristics variables and bone mineral density.

Varieties	All (n=22)					Low (n=11)					High (n=11)				
	TB	Arm	Spine	Pelvis	Leg	TB	Arm	Spine	Pelvis	Leg	TB	Arm	Spine	Pelvis	Leg
Age	−0.44 *	−0.60 **	−0.28	−0.37	−0.51 *	−0.21	−0.33	−0.11	−0.41	−0.31	−0.74 **	−0.79 **	−0.47	−0.42	−0.75 **
BMI	0.39	0.37	0.46 *	0.49 *	0.42	0.34	0.18	0.33	0.30	0.42	0.41	0.48	0.57	0.59	0.39
SMI	0.12	0.16	−0.03	−0.04	0.34	0.16	0.05	−0.09	−0.12	0.18	0.06	0.19	−0.05	−0.08	0.15
Body fat	0.27	0.23	0.42	0.52 *	0.18	0.31	0.23	0.40	0.40	0.44	0.20	0.21	0.42	0.56	0.25
Atherosclerosis parameter															
cf-PWV	−0.23	−0.03	−0.03	−0.14	−0.34	−0.01	0.18	0.22	0.41	0.11	0.37	0.24	0.47	0.24	−0.04
ba-PWV	−0.02	0.11	0.29	0.14	−0.20	0.33	0.38	0.58	0.54	0.32	0.30	0.24	0.63	0.46	−0.05
SBP	−0.10	−0.01	0.11	−0.14	−0.28	0.28	0.37	0.57	0.46	0.33	0.17	0.00	0.23	−0.07	−0.23
DBP	0.04	0.19	0.19	0.05	-0.03	0.19	0.24	0.41	0.42	0.27	0.53	0.47	0.48	0.28	0.31
Biochemical parameter															
Total cholesterol	0.17	0.26	0.44 *	0.39	0.14	0.23	0.34	0.51	0.23	0.21	0.32	0.29	0.54	0.73 *	0.28
Triglyceride	0.09	0.09	0.21	0.29	0.12	−0.34	−0.47	−0.26	−0.29	−0.27	0.50	0.46	0.66 *	0.71 *	0.46
HDL cholesterol	−0.09	0.03	−0.13	−0.18	−0.03	−0.26	−0.04	−0.26	−0.38	−0.19	0.11	0.10	0.04	−0.02	0.13
LDL cholesterol	0.21	0.24	0.50 *	0.45 *	0.14	0.44	0.45	0.72 *	0.50	0.37	0.15	0.13	0.39	0.60 *	0.11
HDL percentage	−0.14	−0.09	−0.35	−0.34	−0.08	−0.31	−0.17	−0.50	−0.40	−0.25	−0.07	−0.05	−0.26	−0.41	−0.02
Blood sugar	−0.27	−0.15	−0.18	−0.21	−0.16	0.00	−0.04	−0.03	−0.19	0.12	−0.35	−0.16	−0.21	−0.15	−0.18
HbA1C	−0.27	−0.31	−0.12	−0.36	−0.45 *	−0.33	−0.40	−0.09	−0.44	−0.46	−0.08	−0.22	−0.03	−0.21	−0.34
Accelerometry parameter															
Total MVPA	−0.19	−0.15	0.00	0.04	−0.03	−0.52	−0.40	−0.29	−0.50	−0.39	−0.06	−0.01	0.24	0.39	0.11
Locomotive	0.10	0.18	0.11	0.18	0.34	0.39	0.43	0.47	0.40	0.55	−0.22	−0.01	−0.25	−0.02	0.15
Non-Locomotive	−0.24	−0.23	−0.04	−0.02	−0.16	−0.64 *	−0.54	−0.43	−0.63 *	−0.56	0.05	0.00	0.42	0.48	0.04
Steps	−0.01	−0.01	0.01	0.10	0.20	0.20	0.03	0.34	0.22	0.15	−0.20	−0.06	−0.24	0.02	0.22
Nutritional parameters ^a^															
Protein	−0.15	−0.26	−0.15	−0.15	−0.08	0.02	−0.18	−0.15	−0.15	0.08	−0.51	−0.42	−0.15	−0.32	−0.37
Fat	0.21	0.28	0.18	0.18	0.18	−0.10	-0.11	0.18	0.18	-0.17	0.53	0.35	0.18	0.45	0.39
SFA	0.15	0.27	0.11	0.11	0.15	−0.08	−0.04	0.11	0.11	−0.17	0.42	0.28	0.11	0.35	0.37
MUFA	0.22	0.31	0.21	0.21	0.19	−0.10	−0.04	0.21	0.21	-0.13	0.63	0.47	0.21	0.55	0.45
PUFA	0.20	0.11	0.18	0.18	0.12	−0.07	−0.18	0.18	0.18	-0.15	0.34	0.21	0.18	0.31	0.18
n-3 fatty acids	0.09	−0.14	0.07	0.07	−0.01	0.15	−0.13	0.07	0.07	0.05	0.01	−0.05	0.07	−0.02	−0.06
n-6 fatty acids	0.20	0.18	0.18	0.18	0.14	−0.14	−0.16	0.18	0.18	−0.19	0.41	0.27	0.18	0.38	0.24
Carbohydrate	−0.04	0.10	−0.01	−0.01	0.03	0.00	0.15	−0.01	−0.01	0.07	−0.24	−0.11	−0.01	−0.23	−0.17
Sodium	-0.21	-0.38	-0.16	−0.16	−0.21	0.02	−0.23	−0.16	−0.16	0.03	−0.47	−0.39	−0.16	−0.33	−0.39
Potassium	0.16	0.06	0.12	0.12	0.20	0.25	0.09	0.12	0.12	0.37	−0.12	−0.08	0.12	−0.02	−0.15
Calcium	0.15	0.01	0.10	0.10	0.20	0.23	0.03	0.10	0.10	0.28	−0.05	−0.10	0.10	−0.01	0.03
Magnesium	0.00	−0.14	−0.02	−0.02	0.05	0.17	0.00	−0.02	−0.02	0.28	−0.39	−0.32	−0.02	−0.24	−0.34
Phosphorus	−0.15	−0.26	−0.15	−0.15	−0.08	0.02	−0.18	−0.15	−0.15	0.08	−0.51	−0.42	−0.15	−0.32	−0.37
Iron	−0.13	−0.27	−0.18	−0.18	−0.05	−0.05	−0.23	−0.18	−0.18	0.06	−0.52	−0.42	−0.18	−0.41	−0.41
Zinc	−0.27	−0.31	−0.18	−0.18	−0.15	−0.18	−0.26	−0.18	−0.18	0.01	−0.46	−0.32	−0.18	−0.17	−0.35
Copper	−0.15	−0.27	−0.15	−0.15	−0.10	−0.01	−0.17	−0.15	−0.15	0.10	−0.48	−0.37	−0.15	−0.30	−0.41
Manganese	−0.51 *	−0.57 **	−0.48 **	−0.48 *	−0.43	−0.18	−0.46	−0.48	−0.48	−0.29	−0.74 **	−0.58	−0.48	−0.65 *	−0.48
β-carotene eq	0.19	0.10	0.13	0.13	0.26	0.09	−0.01	0.13	0.13	0.30	0.00	0.06	0.13	−0.02	−0.11
Vitamin D	−0.02	−0.22	−0.04	−0.04	−0.01	0.17	−0.06	−0.04	−0.04	0.18	−0.61 *	−0.49	−0.04	−0.52	−0.46
α-tocopherol eq	0.30	0.21	0.19	0.19	0.33	0.05	−0.06	0.19	0.19	0.21	0.27	0.18	0.19	0.15	0.13
Vitamin K	0.06	−0.04	−0.01	−0.01	0.13	−0.02	−0.16	−0.01	−0.01	0.12	−0.21	−0.13	−0.01	−0.13	−0.20
Vitamin B1	0.02	0.00	0.02	0.02	0.09	0.11	0.05	0.02	0.02	0.35	−0.04	0.00	0.02	0.07	−0.10
Vitamin B2	−0.17	−0.28	−0.20	−0.20	−0.09	−0.12	−0.34	−0.20	−0.20	−0.12	−0.36	−0.34	−0.20	−0.25	−0.20
Niacin	−0.06	−0.20	−0.07	−0.07	−0.04	0.10	−0.13	−0.07	−0.07	0.12	−0.42	−0.31	−0.07	−0.26	−0.34
Vitamin B6	0.04	−0.07	0.00	0.00	0.07	0.14	−0.04	0.00	0.00	0.23	−0.18	−0.09	0.00	−0.09	−0.17
Vitamin B12	−0.10	−0.28	−0.12	−0.12	−0.10	0.05	−0.21	−0.12	−0.12	0.00	−0.50	−0.36	−0.12	−0.40	−0.34
Folic acid	0.05	−0.06	−0.01	−0.01	0.14	0.15	−0.02	−0.01	−0.01	0.30	−0.37	−0.26	−0.01	−0.36	−0.30
Pantothenic acid	−0.27	−0.31	−0.18	−0.18	−0.15	−0.18	−0.26	−0.18	−0.18	0.01	−0.46	−0.32	−0.18	−0.17	−0.35
Vitamin C	0.23	0.14	0.16	0.16	0.30	0.32	0.19	0.16	0.16	0.46	−0.14	−0.09	0.16	−0.12	−0.10
Suger	0.30	0.50 *	0.15	0.15	0.40	0.34	0.59	0.15	0.15	0.40	0.02	0.01	0.15	−0.31	0.14
Alcohol	−0.02	−0.18	−0.04	−0.04	−0.09	0.13	0.07	−0.04	−0.04	0.09	0.16	0.07	−0.04	−0.18	0.10
Genistein	−0.20	−0.33	−0.17	−0.17	−0.19	−0.13	−0.30	−0.17	−0.17	−0.18	−0.29	−0.25	−0.17	−0.14	−0.21

TB, total-body; BMI, body mass index; SMI, skeletal mass index; cf-PWV, carotid-femoral pulse wave velocity; ba-PWV, brachial-ankle pulse wave velocity; SBP, systolic blood pressure; DBP, diastolic blood pressure; MVPA, moderate-to-vigorous physical activity; SFA, saturated fatty acid; MUFA, monounsaturated fatty acid; PUFA, polyunsaturated fatty acid; Eq, equivalents. ^a^ All, *n* = 20; Low-PWV, *n* = 9; High-PWV, *n* = 11. Correlation analyses of the relationships between BMD and clinical features. * *p* < 0.05, ** *p* < 0.01.

**Table 5:** Partial correlation coefficients adjusted for age and BMI between physical activity, nutritional parameters and bone mineral density.

Varieties	All (n=22)					Low (n=11)					High (n=11)				
	TB	Arm	Spine	Pelvis	Leg	TB	Arm	Spine	Pelvis	Leg	TB	Arm	Spine	Pelvis	Leg
Accelerometry parameter															
Total MVPA	−0.19	0.02	−0.06	0.06	0.10	−0.59	−0.29	−0.35	−0.40	−0.37	−0.05	0.04	0.12	0.36	0.48
Locomotive	0.00	0.22	−0.09	0.02	0.38	0.29	0.52	0.38	0.41	0.49	−0.39	0.00	−0.63	−0.29	0.41
Non-Locomotive	−0.18	−0.06	−0.02	0.06	−0.03	−0.69 *	−0.44	−0.46	−0.52	−0.51	0.20	0.04	0.50	0.53	0.21
Steps	−0.04	0.06	−0.12	0.04	0.30	0.05	0.05	0.24	0.27	−0.08	−0.12	0.18	−0.37	0.00	0.67
Nutritional parameters ^a^															
Protein	0.05	0.02	−0.16	0.03	0.21	0.06	−0.06	−0.18	−0.06	0.20	−0.09	0.24	−0.19	0.25	0.23
Fat	0.13	0.14	0.18	0.21	0.06	−0.15	−0.18	−0.05	−0.10	−0.25	0.77 *	0.58	0.77 *	0.76 *	0.50
SFA	0.07	0.17	0.08	0.11	0.05	0.00	0.03	0.07	0.00	−0.07	0.37	0.13	0.29	0.45	0.28
MUFA	0.09	0.12	0.18	0.21	0.01	−0.16	−0.16	−0.04	−0.04	−0.24	0.82 **	0.63	0.88 **	0.72 *	0.45
PUFA	0.17	0.01	0.25	0.25	0.05	−0.28	−0.43	−0.17	−0.29	−0.47	0.80 **	0.77 *	0.88 **	0.74 *	0.49
n-3 fatty acids	0.33	0.11	0.29	0.22	0.25	0.13	−0.09	0.08	0.03	0.02	0.66	0.80 **	0.66	0.54	0.51
n-6 fatty acids	0.09	−0.03	0.20	0.23	−0.03	−0.41	−0.50	−0.25	−0.37	−0.60	0.79 *	0.71 *	0.88 **	0.75 *	0.45
Carbohydrate	−0.15	−0.01	−0.10	−0.05	−0.08	0.05	0.13	0.12	0.31	0.14	−0.62	−0.54	−0.56	−0.71 *	−0.48
Sodium	0.07	−0.02	−0.03	0.09	0.16	0.07	−0.10	−0.10	0.06	0.15	0.01	0.34	0.00	0.20	0.19
Potassium	0.21	0.13	0.03	0.09	0.28	0.15	0.08	−0.04	0.05	0.29	0.30	0.53	0.20	0.26	0.24
Calcium	0.26	0.16	0.09	0.17	0.37	0.24	0.17	0.05	0.11	0.36	0.21	0.20	0.13	0.50	0.34
Magnesium	0.10	−0.01	−0.06	0.05	0.20	0.06	−0.02	−0.13	−0.06	0.18	0.17	0.52	0.09	0.38	0.29
Phosphorus	0.05	0.02	−0.16	0.03	0.21	0.06	−0.06	−0.18	−0.06	0.20	−0.09	0.24	−0.19	0.25	0.23
Iron	−0.01	−0.12	−0.23	−0.06	0.14	−0.14	−0.25	−0.35	−0.26	−0.02	−0.04	0.37	−0.16	0.10	0.19
Zinc	−0.16	−0.13	−0.30	0.06	0.04	−0.16	−0.23	−0.41	−0.16	0.06	−0.17	0.26	−0.13	0.23	0.11
Copper	−0.04	−0.13	−0.19	−0.03	0.07	−0.11	−0.22	−0.30	−0.24	−0.01	−0.04	0.37	−0.09	0.10	0.11
Manganese	−0.36	−0.34	−0.42	−0.35	−0.18	−0.14	−0.39	−0.21	−0.16	−0.24	−0.52	−0.08	−0.64	−0.44	0.09
β-carotene eq	0.15	0.03	−0.02	0.09	0.25	−0.04	−0.09	−0.20	0.06	0.17	0.16	0.41	0.21	0.01	−0.04
Vitamin D	0.27	0.13	0.10	0.13	0.36	0.28	0.13	0.08	0.20	0.37	−0.20	0.23	−0.21	0.09	0.11
α-tocopherol eq	0.21	0.03	0.10	0.17	0.24	−0.25	−0.31	−0.30	−0.11	−0.12	0.68 *	0.71 *	0.72 *	0.47	0.36
Vitamin K	0.02	−0.10	−0.14	0.01	0.12	−0.26	−0.34	−0.42	−0.38	−0.16	0.07	0.36	0.10	0.25	0.07
Vitamin B1	0.02	0.02	−0.14	−0.02	0.12	−0.03	−0.04	−0.26	−0.07	0.22	0.28	0.49	0.16	0.14	0.19
Vitamin B2	−0.01	−0.05	−0.18	−0.01	0.15	−0.13	−0.28	−0.31	−0.31	−0.10	0.06	0.24	−0.06	0.40	0.39
Niacin	0.11	0.03	−0.05	0.00	0.18	0.10	−0.06	−0.09	−0.05	0.15	0.08	0.51	0.00	0.15	0.27
Vitamin B6	0.11	0.05	−0.08	−0.07	0.18	0.07	−0.02	−0.13	−0.07	0.19	0.07	0.34	−0.06	−0.07	0.09
Vitamin B12	0.18	0.04	0.01	0.03	0.24	0.15	−0.06	−0.04	−0.03	0.14	−0.14	0.28	−0.14	0.12	0.18
Folic acid	0.11	0.01	−0.09	−0.01	0.25	0.04	−0.04	−0.14	0.04	0.22	−0.09	0.25	−0.18	−0.15	0.04
Pantothenic acid	−0.16	−0.13	−0.30	0.06	0.04	−0.16	−0.23	−0.41	−0.16	0.06	−0.17	0.26	−0.13	0.23	0.11
Vitamin C	0.27	0.20	0.09	0.12	0.39	0.24	0.18	0.06	0.17	0.40	0.19	0.44	0.13	0.12	0.25
Suger	0.13	0.34	0.04	−0.01	0.25	0.34	0.56	0.45	0.45	0.41	−0.33	−0.45	−0.59	−0.63	−0.11
Alcohol	0.05	−0.11	0.03	−0.17	−0.03	0.04	0.06	0.00	−0.26	−0.03	−0.09	−0.38	−0.36	−0.51	−0.24
Genistein	−0.10	−0.23	−0.15	−0.03	−0.08	−0.27	−0.40	−0.34	−0.54	−0.37	0.15	0.35	0.10	0.42	0.31

TB, Total body; cf-PWV, carotid-femoral pulse wave velocity; ba-PWV, brachial-ankle pulse wave velocity; SBP, systolic blood pressure; DBP, diastolic blood pressure; MVPA, moderate-to-vigorous physical activity; SFA, saturated fatty acid; MUFA, monounsaturated fatty acid; PUFA, polyunsaturated fatty acids; Eq, equivalents. ^a^: All *n* = 20, Low *n* = 9, High *n* = 11. * *p* < 0.05, ** *p* < 0.01.
